# Reproductive differentiation into sexual and apomictic polyploid cytotypes in *Potentilla puberula* (Potentilleae, Rosaceae)

**DOI:** 10.1093/aob/mct167

**Published:** 2013-08-19

**Authors:** Ch. Dobeš, A. Milosevic, D. Prohaska, S. Scheffknecht, T. F. Sharbel, K. Hülber

**Affiliations:** 1 Department of Pharmacognosy, Pharmacobotany, University of Vienna, Althanstrasse 14, 1090 Vienna, Austria; 2 Apomixis Research Group, Leibniz-Institute for plant genetics and crop research (IPK), Corrensstrasse 3, D-06466 Gatersleben, Germany; 3 Department of Conservation Biology, Vegetation Ecology and Landscape Ecology, University of Vienna, Rennweg 14, 1030 Vienna, Austria; 4 Vienna Institute for Nature Conservation & Analyses, Giessergasse 6/7, 1090 Vienna, Austria

**Keywords:** Apomixis, endosperm, European Alps, FCSS, flow cytometry, pollen, polyploidy, *Potentilla puberula*, reproductive isolation, Rosaceae, sexual reproduction

## Abstract

**Background and Aims:**

Intraspecific reproductive differentiation into sexual and apomictic cytotypes of differing ploidy is a common phenomenon. However, mechanisms enabling the maintenance of both reproductive modes and integrity of cytotypes in sympatry are as yet poorly understood. This study examined the association of sexual and apomictic seed formation with ploidy as well as gene flow towards sexuals within populations of purely polyploid *Potentilla puberula*.

**Methods:**

The study is based on 22 populations representing various combinations of five polyploid cytotypes (tetraploid–octoploid) from East Tyrol, Austria. Embryo ploidy and the endosperm/embryo ploidy ratio obtained by a flow cytometric seed screen were used to infer reproductive modes of seed formation and to calculate the male and female genomic contributions to the embryo and endosperm. Self-incompatibility (SI) patterns were assessed and a new indirect approach was used to test for the occurrence of intercytotype matings based on the variation in the male genomic contribution to sexually derived embryos on the level of developed seed.

**Key Results:**

Tetraploids formed seeds almost exclusively via sexual reproduction, whereas penta- to octoploids were preferentially apomictic. Non-random distribution of reproductive modes within maternal plants further revealed a tendency to separate the sexual from the apomictic mode among individuals. Self-incompatibility of sexuals indicated functionality of the gametophytic SI system despite tetraploidy of the nuclear genome. We found no indication for significant cross-fertilization of tetraploids by the high polyploids.

**Conclusions:**

The study revealed a rare example of intraspecific differentiation into sexual and apomictic cytotypes at the polyploid level. The integrity of the sexual tetraploids was maintained due to reproductive isolation from the apomictic higher polyploids. Functionality of the gametophytic SI system suggested that the tetraploids are functional diploids.

## INTRODUCTION

Angiosperms show three principal modes of seed formation: regular sexual reproduction involving female meiosis and fertilization of the egg cell, and the asexual modes gametophytic and sporophytic (adventitious embryony) apomixis (Asker and Jerling, 1992; Savidan, 2007). Among the asexual modes, gametophytic apomixis constitutes the prevailing developmental pathway represented in several major plant families including the Asteraceae, Poaceae and Rosaceae (Asker and Jerling, 1992; Carman, 1997). It refers to various pathways involving a female gametophyte (or embryo sac) formed by the modification or loss of meiosis (i.e. apomeiosis), embryo formation usually from an unfertilized egg cell (i.e. parthenogenesis) and the development of the endosperm with (i.e. pseudogamy) or without (i.e. autonomous) fertilization (Nogler, 1984).

Gametophytic apomicts (for convenience we use the term apomicts from hereon) are almost exclusively polyploid (Asker and Jerling, 1992; Carman, 1997) with well-documented exceptions limited to diploid genotypes of *Boechera* (Böcher, 1951) and *Paspalum* (Siena *et al.*, 2008). Apomicts are either allopolyploids – as in most cases [e.g. *Antennaria* (Bayer, 1997); *Potentilla* (Dobeš *et al.*, 2004; Paule *et al.*, 2011)] – or autopolyploids [e.g. *Paspalum* (Hojsgaard *et al.*, 2008); *Ranunculus* (Cosendai *et al.*, 2011); *Townsendia* (Thompson and Whitton, 2006)], whereas their sexual relatives are usually diploid. Concerning the need for excess copies of apomixis factors, polyploidy was proposed to be a requirement for the expression of apomixis (Mogie, 1988). Polyploidy and hybridization may deregulate and repattern gene expression of the normal sexual pathway resulting in apomixis (e.g. Carman, 1997). In accordance with this hypothesis, the sexual ancestors of apomicts are usually outcrossing diploids (Asker and Jerling, 1992). Reproductive differentiation, however, is not necessarily restricted to diploid–polyploid contrasts because it has been rarely observed among polyploids (e.g. Rotreklová *et al.*, 2002).

Space is a crucial factor for the understanding of the evolutionary significance of reproductive differentiation. Sexual and apomictic cytotypes may be spatially separated from each other (i.e. allopatric distribution; e.g. Mráz *et al.*, 2009) or co-occur at the population level (i.e. in sympatry; e.g. Elzinga *et al.*, 1987; Menken *et al.*, 1995; Talent and Dickinson, 2007). Co-existence of reproductively differentiated cytotypes thereby raises questions about mechanisms maintaining their genomic and genetic integrity. The number of (monoploid) genomes *per se* might promote the integrity of cytotypes, since heteroploid crosses frequently show reduced offspring vitality and/or fertility (Ramsey and Schemske, 1998; Hardy *et al.*, 2001). Thus, in the absence of pre-zygotic barriers, sexual individuals differing in ploidy from co-occurring pollen donor plants were shown to be excluded via the minority cytotype exclusion principle (Levin, 1975). However, no cross-fertilization is needed for apomictic embryo development. Consequently, apomicts may not necessarily be suppressed in heteroploid crosses if variable paternal genomic contributions to the endosperm are tolerated (e.g. Kao, 2007) or the endosperm develops autonomously. Furthermore, pollen transferred in heteroploid crosses and pollen of poor quality induced selfing in otherwise self-incompatible sexuals in cross-pollinations (i.e. mentor effects), promoting the genetic integrity and the maintenance of sexuals in the presence of apomictic relatives (Hörandl and Temsch, 2009). In the absence of reproductive barriers isolating sexuals from apomicts, apomixis may swamp sexuals and come to fixation (Mogie, 1992; Adolfsson and Bengtsson, 2007).

Flow cytometry has been established as an effective and reliable tool for the estimation of nuclear DNA contents and DNA ploidy levels (Doležel *et al.*, 2007*b*; Greilhuber *et al.*, 2007). The method is particularly useful for high-density (DNA)ploidy screens of individuals even on fine spatial scales (Suda *et al.*, 2007) as well as for high-throughput reproductive mode seed screening. The latter has been established as a flow cytometric technique called the flow cytometric seed screen (FCSS) (Matzk *et al.*, 2000) and is based on a comparison of DNA content (or DNA ploidy) of the endosperm and embryo. The method differentiates between the meiotic and the apomeiotic formation of the embryo sac, the parthenogenetic and zygotic origin of the embryo, and the autonomous vs. pseudogamous development of the endosperm. Recently, FCSS has been extended to calculate male and female genomic contributions to the embryo and endosperm, respectively, in sexual and pseudogamous apomicts (Dobeš *et al.*, 2013). Calculation of the genomic contribution is based on the assumption that endosperms receive a bi-nucleate female contribution, a prerequisite which first can be proven based on cyto-embryological evidence and (molecular marker-aided) progeny surveys (cf. Dobeš *et al.*, 2013). The mathematical formulae developed by these authors, however, are applicable independently of the origin (meiotic vs. apomeiotic) of the male and female gametophytes and the ploidy of parents, i.e. they allow estimation of gamete ploidy variation caused by meiotic disturbances and intercytotype crosses.

The genus *Potentilla* (Rosaceae) shows considerable variation in reproductive mode among and within species, particularly in the derived and species-rich core group (Dobeš and Paule, 2010; Dobeš *et al.*, 2013). Apomictic elements (i.e. apomeiosis and parthenogenetic origin of embryos) have been documented in at least 16 species (Gentscheff, 1938; Gustafsson, 1947; Löve, 1954; Asker, 1970*a*), while sexual reproduction was claimed for five species based on embryological evidence (Rutishauser, 1945; Håkansson, 1946; Czapik, 1961, 1962*a*). In order to initiate seed formation, in all *Potentilla* species including apomicts functional pollen is needed to fertilize the endosperm (Asker, 1970*b*), which usually receives a bi-nucleate female contribution (Dobeš *et al.*, 2013).

In *Potentilla*, reproductive differentiation into sexuals and apomicts is often associated with extensive variation in ploidy also observed at the population level (e.g. Skalinska and Czapik, 1958; Smith, 1971; Dobeš, 1999; Paule *et al.*, 2011). Despite earlier claims of diploid apomixis in *Potentilla* (Håkansson, 1946; Asker, 1967, 1970*c*), seed formation by diploids appears to be sexual (Holm and Ghatnekar, 1996*b*; Holm *et al.*, 1997; Dobeš *et al.*, 2013), leading to sexual diploid–apomictic polyploid contrasts. However, reproductive differentiation at the polyploid level also seems to exist. In *Potentilla incana* tetraploidy was associated with sexual reproduction (Czapik, 1962*a*) and hexaploidy with apomixis (Ch. Dobeš, unpubl. res.). Tetraploids were sexual in *Potentilla crantzii* (Czapik, 1961, 1962*b*) and *P. tabernaemontani* (= *P. verna*; inclusively *P. puberula*) (Håkansson, 1946), whereas cytotypes of higher ploidy were apomictic in these species (Müntzing, 1931, 1958; Smith, 1963*a*, *b*; Asker, 1985). However, this evidence is based on limited sample sizes, and the generality of the ploidy–reproductive mode distinctions remains uncertain.

*Potentilla puberula* exhibits extensive intra- and interpopulation variability in ploidy (Dobeš, 1999). High frequencies of odd-ploids in natural populations (Dobeš, 1999) and clonal population structure (Paule and Dobeš, 2010) provide indirect evidence for apomixis, whereas the observation of meiotically reduced megaspores suggested sexual reproduction for a tetraploid individual (Håkansson, 1946). However, direct evidence for apomixis and the conclusive proof of sexual reproduction is missing for the species.

Selfing of tetraploid individuals caused a significantly lower seed set (40–100 %) compared with the open-pollinated control in *P. puberula* (Ch. Dobeš, unpubl. res.), suggesting functionality of a self-incompatibility (SI) system. The homomorphic gametophytic SI system is the common type in the Rosaceae (Barrett, 1988; Weller *et al.*, 1995; Sassa *et al.*, 1996), wherein compatibility of the pollen in a cross is determined by the genotype of the male gametophyte and the (sporophytic) genotype of the pollen recipient. A negative correlation between the effectiveness of SI systems and the ploidy level of a species has been reported from the Rosaceae (Dickinson *et al.*, 2007). The generality of this pattern was proposed by Miller and Venable (2000) who observed a breakdown of incompatibility in 92 % of polyploids associated with diploid self-incompatible plants from families known to have gametophytic SI. The phenomenon is explained by the expression of two pollen *S*-alleles in a single pollen grain inhibiting all S-RNases in the style of a flower (Stone, 2002).

In the following, we provide a comprehensive characterization of the reproductive system of five cytotypes of *P. puberula* differing in ploidy, and examine the effects of cytotype mixture on embryo ploidy in sexually derived seed. Specifically, we ask the following questions. (1) Is there an association of reproductive modes with ploidy levels as suggested by the embryological record and the high frequency of odd-ploid cytotypes. (2) Is the gametophytic SI system still functional in sexual polyploids? (3) Do apomictic and particularly odd-ploid individuals expectedly show reduced pollen vitality compared with sexual and/or even-ploids? (4) Are there effective reproductive barriers among cytotypes as suggested by the high frequency of cytologically mixed populations? In particular, are sexual individuals reproductively isolated from apomicts?

## MATERIALS AND METHODS

### Study system

*Potentilla puberula* Krašan (= *P. pusilla* Host, Soják, 2010) belongs to a group of species (*Aureae Vernae sensu* Wolf, 1908) of mainly European distribution which, according to the latest taxonomic treatment, comprises seven sexual and apomictic species (Kurtto *et al.*, 2004). The species exhibits tetraploids (*x* = 7; 2*n* = 28), pentaploids (2*n* = 35), hexaploids (2*n* = 42), heptaploids (2*n* = 49), octoploids (2*n* = 56) and nonaploids (2*n* = 63) in the Eastern Alps (Dobeš, 1999). Except for nonaploids, these cytotypes have been observed within the scope of a ploidy screen of about 2000 individuals from sympatric populations within East Tyrol, Austria (Hülber *et al.*, 2013). Although direct evidence for apomixis is missing for *P. puberula*, embryological studies documented apomixis for its former conspecific *P. tabernaemontani* (Rutishauser, 1943*b*; Smith, 1963*a*). Apomixis was realized in that species as diplospory, i.e. embryo sac mother cells develop from the archespore. Aposporous development of embryo sacs sometimes also occurred side by side with diplospory in the same ovule. The majority of archesporial cells entered a well-defined synapsis condition followed by a complete breakdown of the meiotic process. Egg cells developed parthenogenetically. Mostly two polar nuclei lying close to each other were observed and fused to form the central cell nucleus.

### Plant material

A total of 115 individuals of known ploidy (determined by Hülber *et al.* 2013), covering one to three ploidy levels in each of the 22 sampled populations, were included in the study (Table 1). To uncover reproductive modes of seed formation, field-collected mature seeds sampled from the same plants in 2010 were stored in paper bags until analysed flow cytometrically in spring 2012. Plants were transplanted to the experimental garden of the Department of Pharmacognosy, University of Vienna and grown in pots (14 cm in diameter) using a substrate composed of six parts ground soil, two parts of bark humus and two parts of quartz sand. We used flowers of these plants to determine pollen quality in the following year.

**Table 1.** General description of 22 populations of *Potentilla puberula* in East Tyrol, Austria, including the geographic origin and pathways of seed formation obtained using the flow cytometric seed screen (FCSS) classified by the ploidy of the maternal plant

Geographic origin					FCSS analysis								
ID	Population	Latitude	Longitude	m a.s.l	Ploidy	*N* specimen	*N* seeds	Reproductive pathway					*N* failed
								Apo	Sex	*L*	*Z*	Unknown	
1	Gonzach	46·87578	12·66265	870	5*x*	3	12	9					3
					6*x*	4	20	10	2	1		6	1
2	Unterleibnig	46·90337	12·63542	805	5*x*	4	13	12				1	
					6*x*	1	5	2				3	
3	Außer Klaunzer-Berg	46·97385	12·55678	1100	5*x*	3	13	9				1	3
					7*x*	1	5	3				2	
4	Oberpeischlach	46·93583	12·59405	1090	5*x*	2	9	6				2	1
					7*x*	1	5	3				2	
5	Rabenstein	47·00903	12·46575	1360	5*x*	4	18	14			1	2	1
6	Obermauern	47·00472	12·43544	1320	4*x*	3	13		11			2	
					6*x*	1	4		3			3	
7	Hainfels	46·75068	12·43715	1190	4*x*	5	20		17			2	1
					7*x*	4	17	13				3	1
8	Bobojach	47·01700	12·40368	1390	4*x*	5	15		15				
					5*x*	1	5	4				1	
9	Raut	46·78112	12·57448	1470	4*x*	4	12		12				
					5*x*	1	4	4					
10	Zabernig	47·00467	12·5192	1330	4*x*	3	11		10			1	
					5*x*	4	12	11					1
11	Kosten	47·01883	12·33275	1450	5*x*	3	9	9					
					8*x*	2	10	6				1	3
12	Hopfgarten	46·78628	12·60243	1250	5*x*	4	14	12				2	
					7*x*	1	3	3					
13	Groder	46·92607	12·52558	1530	4*x*	4	14	1	12			1	
					5*x*	3	9	9					
14	Erlbach	46·74653	12·36964	1290	5*x*	2	6	6					
					7*x*	3	11	8	1			2	
					8*x*	7	34	18	1			13	2
15	Lana	46·98575	12·63190	1320	5*x*	3	9	9					
					6*x*	3	9	9					
					8*x*	1	4	3				1	
16	St. Veit	46·92663	12·42213	1580	5*x*	3	9	9					
17	Stein	47·02757	12·52672	1350	5*x*	3	12	7		1		4	
					7*x*	2	8	4	3			1	
					8*x*	3	12	8				4	
18	Innervillgraten	46·81183	12·36085	1450	5*x*	1	3	3					
					8*x*	1	4	4					
20	Dorfmäder	47·02528	12·36367	1720	4*x*	5	15		15				
21	Moaalm	47·03358	12·62811	1800	5*x*	1	3	3					
					6*x*	1	4	4					
22	Katalalm	47·05761	12·48822	1720	5*x*	3	9	9					
38	Obergaimberg	46·84612	12·78215	930	7*x*	2	7	5	1			1	

Latitude/longitude are provided in WGS84 standard. 4*x*, 5*x*, 6*x*, 7*x* and 8*x* refer to tetra-, penta-, hexa, hepta- and octoploids, respectively. ‘*N* specimen’ and ‘*N* seeds’ specify the number of individuals and seeds used in the FCSS, respectively. ‘Reproductive pathways’ refers to the apomictic (Apo) and sexual (Sex) origin of seeds. Following Dobeš *et al.* (2013), ‘*L*’ indicates fertilization of an unreduced egg cell and ‘*Z*’ indicates apomixis involving an embryo sac of twice the ploidy of the maternal plant. ‘Unknown’ indicates seeds missing a distinct fluorescence signal for the endosperm. ‘*N* failed’ is the number of seeds which failed in the FCSS.

### Flow cytometric seed screen (FCSS)

The relative fluorescence intensity of embryo and endosperm nuclei was determined by flow cytometric analysis of single seeds following Dobeš *et al.* (2013). Three to five seeds were analysed per individual, 432 seeds in total. *Pisum sativum* ‘Kleine Rheinländerin’ and *Glycine max* ‘Inovec’ (Doležel *et al.*, 2007*a*) were chopped together with the sample and served as internal standards. 4',6-Diamidino-2-phenylindole (DAPI) served as the DNA-selective stain. Measurements were performed on a CyFlow Ploidy Analyser equipped with a 365 nm light-emitting diode (LED Partec, Germany). The sample/standard fluorescence ratio and the endosperm/embryo fluorescence ratio (i.e. the peak index) were calculated from the means of the corresponding fluorescence histograms. The DNA ploidy of embryos was inferred from comparison of the sample/standard fluorescence ratio of seeds with the sample/standard fluorescence ratio of reference individuals of known chromosome number (Ptl4048, 2*n* = 4*x* = 28; Ptl4184, 2*n* = 5*x* = 35; Ptl4187, Ptl4188, 2*n* = 7*x* = 49: Paule *et al.*, 2012). For convenience, we refer to the measured DNA ploidy (Suda *et al.*, 2006) as ploidy.

### Inference of reproductive modes and of the male and female genomic contribution

We distinguish between the sexual and the apomictic origin of the embryo. Peak indices <2 are indicative of a sexual origin, while values >2 in combination with the recovery of the maternal ploidy by the embryo indicate apomixis. The female and male genomic contributions are calculated from the embryo and endosperm ploidies using the mathematical formulae introduced by Dobeš *et al.* (2013). The female genomic contribution to the embryo and endosperm is once and twice the ploidy of the embryo sac, respectively. The male genomic contribution is the number of male genomes transferred by the two sperm to the embryo and endosperm in seeds with sexually derived embryos and by one or two sperm to the endosperm in seeds with parthenogenetically derived embryos. We use, according to Greilhuber (2005), *n* (the haplophasic chromosome number) to indicate the number of holoploid genomes (i.e. the whole chromosome complement with chromosome number *n*) and *x* (the chromosome number of the monoploid genome) when referring to the number of chromosome sets (i.e. the generative ploidy). The female genomic contribution is provided as *n* or *x*. The male genomic contribution is calculated as *x* only because *n* of the pollen donor is unknown. Estimates of male and female genomic contributions were used to calculate maternal : paternal genome ratios in the endosperm.

To test whether sex and apomixis were randomly associated with each other in a single maternal plant, we applied a Monte Carlo randomization technique using R (R Development Core Team, 2011). The empirical association of reproductive modes was compared with the distribution of associations of 10 000 replications randomly assigning modes to seeds. As a measure of association, we used the percentage of individuals with at least one sexual seed which also derived at least one seed via apomixis – and vice versa.

The effect of population cytotype diversity on the male genomic contribution to the embryo was tested using linear regressions performed for sexually derived seeds of tetraploids. Diversity and the male genomic contribution were measured as the Shannon diversity index based on the cytological diversity of populations (Supplementary Data Table S1) and as *x*, respectively. Analyses were performed using R (R Development Core Team, 2011).

### Breeding system of sexuals

We combined controlled pollinations and the determination of pollen/ovule (P/O) ratios (Cruden, 1977) to infer the breeding system of sexuals. Pollination experiments were carried out from March to May 2011. Flowers were emasculated and bagged a few days before anthesis. Bridal veil was used for bagging as it has the least effect on the microclimate of the bagged flowers (Wyatt *et al.*, 1992). At stigma maturity, flowers were selfed and outcrossed, respectively, by rubbing mature anthers over the recipient stigmas. Each treatment was applied to two flowers of each of 38 individuals. At seed maturity, the number of viable seeds and empty testae was assessed, enabling the calculation of seed/ovule and P/O ratios of each flower. We performed pairwise reciprocal cross-pollinations with all individuals from two randomly selected populations inhabited by tetraploids (populations 6 and 13). The number of filled seed was compared as a measure of the reproductive success between selfed and outcrossed flowers using a generalized linear model. We assumed the number of seeds to be a Poisson-distributed random variable and, thus, applied a log-link function. To consider potential autocorrelation of values derived from flowers of the same individual, we included treatment as a random effect for each pollen receptor plant. The analysis was performed using the function glmer of the library lme4 (Douglas Bates, Martin Maechler and Ben Bolker, 2012. lme4: Linear mixed-effects models using S4 classes. R package version 0·999999-0. http://CRAN.R-project.org/package=lme4#) in R (R Development Core Team, 2011).

The P/O ratios were estimated for a single flower per individual. Anthers were preserved in Carnoy's fixative (60 % ethanol:30 % chloroform:10 % acetic acid) and stained with a solution of Malachite green, acid fuchsin and Orange G (Peterson *et al.*, 2010) for approx. 12 h. Subsequently one mature undehiscent anther per flower was transferred to a glass slide and covered in a drop of 100 µL of distilled water, finely chopped with a razor blade and the resulting suspension homogenized. A 20 µL aliquot of the suspension was transferred to a Fuchs Rosenthal counting chamber (Hecht Assistent, Altau, Switzerland) and pollen grains were counted using a light microscope (Nikon Eclipse 600, Nikon, Japan). Ovules per flower were counted with the aid of a stereo lens (Nikon SMZ-U, Nikon, Japan).

### Estimation of pollen quality

Pollen quality was estimated based on the percentage of physiologically vital and morphologically intact pollen grains of a single anther per individual. Anthers were stained using the vitality stain invented by Peterson *et al.* (2010), which discriminates aborted from non-aborted pollen based on the stainability of the protoplasm. In addition, the shape of pollen grains was used to discriminate morphologically intact pollen (regularly round to oval) from degraded (i.e. deformed) pollen. Only stained and morphologically regular grains were regarded as viable. Pollen was embedded in a drop of distilled water dispersed between an object and cover slide, and 94–211 pollen grains per individual were screened for their viability using a Nikon Eclipse 600 light microscope and bright-field illumination.

Differences in pollen viability among ploidy levels were tested by means of logistic regressions using the proportion of viable pollen grains as response and ploidy level as a categorical predictor. The number of individuals was used as a weighting factor, because proportions of viable pollen were pooled over individuals for each cytotype within populations. In regression analyses, categorical predictors such as ploidy allow for pairwise comparisons only with a pre-defined baseline level. Thus, it was necessary to re-fit the model using different cytotypes as baseline levels, i.e. each cytotype was compared with the remaining ones in a separate model. An inflation of Type I errors due to multiple comparisons was avoided by applying a Bonferroni correction of resulting *P*-values. Analyses were performed using R (R Development Core Team, 2011).

## RESULTS

### Variation in reproductive mode

Clear fluorescence signals for both the embryo and the endosperm were obtained from 354 (81·94 %) seeds using FCSS. The remaining seeds either failed (3·93 %) or showed signals for the embryo only (14·13 %). A total of 102 (28·81 %) of the seeds with embryo and endosperm signals were derived through regular sexual reproduction (i.e. fertilization of the reduced egg cell), while 249 seeds (70·34 %) were of apomictic origin. Two seeds originated from the fertilization of an unreduced egg cell (exemplary measurements graphically representing these modes are provided in Supplementary Data Fig. S3). The ploidy of the maternal plant (2*n* = 5*x*, population 5) was doubled in a single apomictically derived embryo (4*n* = 10*x*). The association of inferred reproductive pathways and modes of seed formation with the ploidy of maternal plants is shown in Table 2. Tetraploids formed 98·9 % of their seeds via regular sexual reproduction. In contrast, higher polyploids (penta- to octoploids) were preferentially apomictic (88·6–100 % of the analysed seeds depending on cytotype). Seven out of the 115 maternal plants formed seeds via both apomixis and sexual reproduction. This share was significantly lower (Monte Carlo randomization: *P* < 0·001 in both cases) than expected for a random association of reproductive modes based on the observed frequencies of reproductive modes and, thus, shows some tendency to separate the sexual from the apomictic modes among individuals. In nine out of the 22 populations, sexually as well as apomictically derived seeds were found. In contrast, sexual reproduction and apomixis only were observed in two and 11 populations, respectively. A detailed description of the FCSS results is given in the Supplementary Data Results.

**Table 2.** Reproductive modes of seed development observed in five cytotypes of *Potentilla puberula* (Rosaceae)

Ploidy of the maternal plant	Sexual	Apomictic	Irregular
Tetraploid	92	1	0
Pentaploid	0	145	2
Hexaploid	4	25	1
Heptaploid	5	39	0
Octoploid	1	39	0

Three to five seeds of each of 115 maternal plants were analysed using flow cytometric seed screen (FCSS).

### The male and female genomic contribution

The male genomic contribution was related to the reproductive mode. Endosperms and embryos derived via sexual reproduction received a male genomic contribution of 1·57*x* to 4·06*x* (Supplementary Data Fig. S2). The female genomic contribution to sexually derived embryos ranged between 0·88*n* and 1·20*n*. In the tetraploids, the male and female genomic contribution to sexually derived embryos varied between 1·57*x* and 2·24*x*, and 1·78*x* and 2·39*x*, respectively. The contributions were negatively correlated with each other (*r*^2^ = 0·755, *P* < 0·001; not significant for the other cytotypes) and resulted in embryo ploidies of 3·87–4·17*x*. The ratio of the female (maternal *m*) to male (paternal *p*) genomic contribution to the endosperm for sexually derived seeds was 2*m*:0·7–1·4*p*. The male genomic contribution to the endosperm in apomictically derived seeds was greatly raised compared with sexual seed and varied between 1·71*x* and 15·74*x* (Supplementary Data Fig. S2). The female genomic contribution to apomictically derived embryos equals by definition the ploidy of the embryo (see the Materials and Methods). The ratio of the female to male genomic contribution to the endosperm was 2*m*:0·3–2·2*p*.

Linear regressions (*F*_1,5_ = 3·96, *P* = 0·103, *R*^2^ = 0·44) revealed no significant relationship of the cytotype diversity of populations (Shannon diversity index) to the male genomic contribution to the embryo (and endosperm) of sexually derived seeds of tetraploids (Supplementary Data Fig. S4).

### Breeding system of sexuals

The breeding system was established for tetraploid sexual individuals. The proportion of flowers with at least one viable seed was 9·3 % and 65·5 % for selfed and outcrossed individuals, respectively. A generalized linear model revealed a significantly higher number of viable seeds for outcrossed than for selfed flowers: fixed effect coefficient ± s.e. = 5·83 ± 1·03; *z* = 5·63; *P* < 0·001; number of groups (i.e. pollinated plants) = 38; number of observations (i.e. flowers) = 484. The P/O ratio ± s.d was 7288·92 ± 5018·08. Thus, tetraploids can be classified as obligate outcrossers following the classification of Cruden (1977).

### Pollen quality

**Fig. 1.**
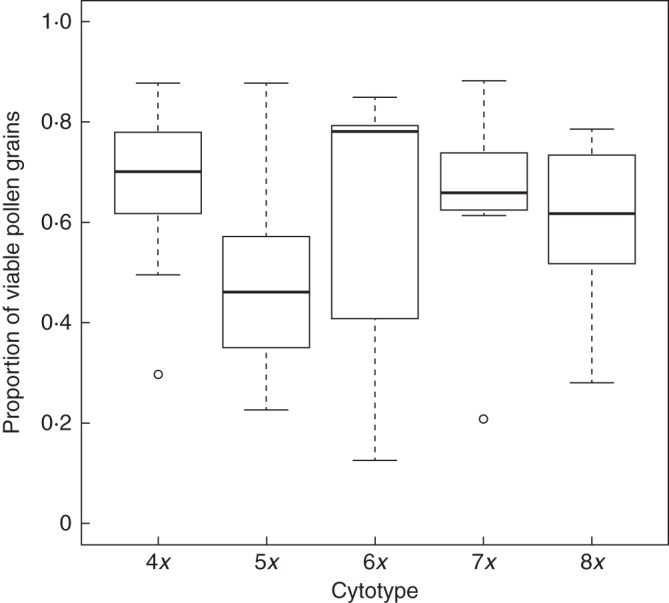
Pollen viability of the five cytotypes of *Potentilla puberula*. Values represent the proportion of viable grains pooled over individuals of each cytoypes in each population. Tetra- (4*x*), penta- (5*x*), hexa- (6*x*), hepta- (7*x*) and octoploids (8*x*) are represented by nine, 22, five, 11, and six populations, respectively.

Pollen quality varied greatly among individuals, from (almost) complete failure to very high percentages of viable pollen in all cytotypes (1·0–99·1 %, 0·0–92·9 %, 12·6–96·9 %, 0·0–88·1 % and 0·0–86·5 % for tetra-, penta-, hexa-, hepta- and octoploid individuals, respectively). The proportion of viable pollen pooled within populations (Fig. 1) differed significantly among cytotypes of *P. puberula* (*P* < 0·001 for all pairwise comparisons; Supplementary Data Table S2). The highest pollen quality was detected in tetraploids, followed by hepta-, hexa-, octo- and pentaploids.

## DISCUSSION

### Apomixis and sexual reproduction are non-randomly associated on the level of the individual

Regular sexual reproduction and apomixis were the dominant modes of seed formation in *P. puberula* (observed for 28·81 and 70·34 % of seeds, respectively). These reproductive modes were non-randomly associated on the level of the individual, as sexually and apomictically derived seeds had a much lower probability of co-occurrence within a single individual than can be expected based on their frequencies (Monte Carlo randomization: *P* < 0·001). Based on the observed relative frequencies of sex and apomixis and under the hypothetical assumption of random association of modes, the probability of an individual to form its seeds – from three to five according to our sampling design – exclusively via one mode is 0·2–2·4 % (sex) and 17·2–34·8 % (apomixis). Actually, 93·9 % of the individuals formed all analysed seeds via a single reproductive mode. This value is expected (based on our sampling) if seeds have a probability of 98·8–97·9 % to form either via sexuality or apomixis. Thus, our data indicated a tendency of individuals to produce their seeds via either the one or the other mode. Our sampling design, however, precluded a classification of individuals as obligate or facultative apomicts/sexuals. Nevertheless, this finding is concordant with high levels of either apomixis or sexuality found in individuals from various *Potentilla* species. On the one hand, high degrees of apomixis were observed in numerous embryological studies (e.g. Rutishauser, 1943*a*; Smith, 1963*a*; Asker, 1970*b*), progeny surveys (Müntzing, 1928; Holm and Ghatnekar, 1996*a*) and an FCSS-based study (Dobeš *et al.*, 2013). On the other hand, sexual reproduction but no or only marginal frequencies of apomixis were detected based on segregation patterns of isozyme markers and FCSS in individuals of *P. argentea* (Holm *et al.*, 1997) and other *Potentilla* species (Dobeš *et al.*, 2013), respectively. Furthermore, histoembryological studies substantiated high degrees of sexual reproduction in *Potentilla*, but available analyses are restricted to few individuals (Håkansson, 1946; Czapik, 1961, 1962*a*).

### Strong reproductive differentiation between tetraploid and high polyploid cytotypes parallels diploid–polyploid systems

Tetraploid *P. puberula* were mainly sexual, whereas penta-, hexa-, hepta- and octoploids preferentially reproduced via apomixis. As already discussed, for numerous angiosperms, intraspecific reproductive differentiation among ploidy levels has been established. Commonly, diploid cytotypes are sexual while polyploids are apomictic (Yahara, 1990; Savidan *et al.*, 2001; Thompson and Whitton, 2006; Lo *et al.*, 2009; Mráz *et al.*, 2009; Cosendai *et al.*, 2011). In contrast, intraspecific differentiation into sexual and apomictic polyploid cytotypes is a rare situation. In embryological studies, association of tetraploidy and high polyploidy with sexuality and apomixis, respectively, was found for *P. crantzii*, *P. incana* and *P. tabernaemontani* (Müntzing, 1931; Håkansson, 1946; Müntzing, 1958; Czapik, 1961, 1962*b*; Smith, 1963*b*; Asker, 1985), suggesting some degree of generality of this pattern in the genus *Potentilla*. However, to the best of our knowledge, differentiation into sexual and apomictic cytotypes on the polyploid level from outside the genus *Potentilla* is documented only for two genera: three *Paspalum* species (Savidan *et al.*, 2001) and *Pilosella officinarum* (Rotreklová *et al.*, 2002). These examples and our study system show important parallels to those involving diploids. Individuals of the lowest ploidy level (i.e. either tetraploids or diploids) are sexual and self-incompatible (Savidan, 2001; Mráz, 2008; Hörandl, 2010; Prohaska, 2013). Furthermore, an allopolyploid origin was suggested for both *P. officinarum* (Mráz *et al.*, 2008) and *P. puberula* (Wolf, 1908; Ehrendorfer, 1970; Soják, 2010). In allotetraploids, inheritance patterns are disomic in most cases because only chromosomes from the same parental species are able to pair in meiosis (Soltis and Soltis, 2009). We have no empirical data on the mode of inheritance or the genetic organization of the tetraploid *P. puberula* genome. However, cytological and genetic diploidization of the genome may be assumed because only functionally disomic incompatibility loci are likely to survive in a polyploid (Richards, 1997). Hence, functionality of the SI system in *P. puberula* may indicate that the tetraploids are functional diploids, as suggested by Mráz *et al.* (2008) for *P. officinarum*.

### Reproductive inter-relationships among cytotypes

Besides reproductively uniform populations (two sexual and 11 apomictic), we found both reproductive modes coexisting in varying proportions in nine populations involving different apomictic cytotypes (Table 1). The presence of heteroploid pollen donors potentially fosters changes in ploidy from the maternal plant to sexually derived embryos due to the possibility for intercytotype pollination. In cases where seeds develop from intercytotype crosses, the variability in endosperm ploidies might be related to the cytological diversity of populations in both sexually and apomictically derived seeds. However, we did not find a correlation between cytological diversity and the male genomic contribution to the embryo and the endosperm in sexual tetraploid *P. puberula* (Supplementary Data Fig. S4). The result is supported by the ploidy of embryos and of involved gametes: the measured ploidy of sexually derived embryos deviated only slightly from 4*x* (3·87–4·17*x*), the maternal ploidy. The relatively higher variation in gamete ploidies (1·57–2·24*x* and 1·78–2·39*x* for the male and female genomic contributions, respectively) contributing to the tetraploid embryos is considered a mathematical artefact because the male genomic contribution is calculated as the difference between the ploidy of the embryo and the female genomic contribution to the embryo (Dobeš *et al.*, 2013). The dependence is seen from the strongly negative correlation between the male and female genomic contributions (Supplementary Data Fig. S2). Hence, gamete ploidies – and resultant embryo ploidies – suggested that for the tetraploid cytotype, sexually derived seeds originated from intracytotype crosses or selfing. Thus, we found no indication for extensive intercytotype gene flow towards the tetraploids on the level of seeds, suggesting integrity through generations of this cytotype in cytologically mixed populations.

Understanding reproductive relationships in apomictic *P. puberula* is complicated by the high variation in the male genomic contribution to the endosperm observed for all high polyploid cytotypes (Supplementary Data Fig. S2), which can be explained by – in addition to intercytotype cross-fertilization of the central cell – irregular meiotic segregation of chromosomes, the methodological error of the inference process and particularly the contribution of either one or two sperm nuclei to the endosperm. Comparable variability in the male genomic contribution (0·35–1·9*n*) to the endosperm of apomictically derived seeds was observed in the offspring of selfed *Potentilla* individuals (Dobeš *et al.*, 2013), suggesting that seed formation in apomictic *P. puberula* likewise may have originated from selfings or intracytotype crosses. However, we cannot distinguish between the effect of intercytotype crosses, i.e. variable sperm ploidy, and that of variation in the number of sperm nuclei on the variability of endosperm ploidy in apomictically derived seeds. Hence, more comprehensive experimental investigations are necessary to disentangle these factors accurately.

Taken together, we found no indication for extensive cross-fertilization of tetraploids. Consequently, effective barriers to gene flow must exist in natural populations of *P. puberula*. On theoretical grounds, barriers might be pre-zygotic (e.g. spatial clustering of cytotypes, ecological differentiation or pollinator preferences, etc.) and/or post-zygotic (e.g. pollen competition, endosperm incompatibilities, etc.). The actual factors maintaining the integrity of cytotypes are not known yet. In addition, the effects of cytotype mixture on seed set and fertility and therefore stability of cytotype mixtures remain to be studied. However, preliminary results from controlled heteroploid crossings show maternal:paternal genome ratios to vary considerably for both sexually (maternal:paternal genome ratios 2:1 to 2:3·4) and apomictically (2:0·6 to 2:4·4) derived seed, indicating a strong relaxation of genomic endosperm balance requirements (Scheffknecht *et al.*, 2013). Furthermore, ecological niche differentiation among tetraploids and high polyploids (Hülber *et al.*, 2013) suggests pre-zygotic isolation of cytotypes to be more important.

### Pollen quality is weakly associated with reproductive mode

Each of the five cytotypes displayed a wide range (in common 0·0–96·9 % of viable pollen grains; Fig. 1) of pollen quality. The low pollen viability observed in some tetraploid individuals is in contrast to the results from other sexual *Potentilla* species showing (close to) 100 % viable pollen (Müntzing, 1928; Czapik, 1961). Lower pollen fertility (53·0–91·5 % viable pollen) was reported from sexual *P. arenaria*, but the embryology of the studied biotypes exhibited some tendency to apomixis (Czapik, 1962*a*). A low frequency of apomixis was also observed in tetraploid *P. puberula* (one out of 93 seeds). Reduced pollen quality in apomicts compared with sexuals was observed for several *Potentilla* species [e.g. approx. 15–80 % viable pollen in *P. tabernaemontani* (Müntzing, 1928; Asker, 1985); approx 40–60 % in *P. argentea*, approx. 0–40 % in *P. collina* (Müntzing, 1928, 1958); and 64 % in *P. intermedia* (Asker, 1970*a*)]. Besides apomixis, hybridity was associated with low pollen quality (Müntzing, 1928; Asker, 1970*a*). Thus, the low pollen quality in some tetraploid *P. puberula* individuals might be the combined effects of a tendency to apomixis and the hybrid origin of the species.

In almost all cases documented in *Potentilla* species, poor pollen quality was linked to disturbances of male meiosis, indicated by irregular chromosome pairing, laggards, sticking chromosome bridges, microcyte formation or degeneration of nuclei (e.g. Müntzing, 1928; Asker, 1970*a*; Czapik, 1975), resulting in aneuploid offspring (Asker, 1971). Such irregularities can be particularly expected in odd-ploids because of the high chance of unpaired chromosomes disrupting meiosis (Dawe, 1998). However, high pollen quality was found in heptaploid *P. puberula*, which might be accomplished by the formation of unreduced male gametes (e.g. Voigt *et al.*, 2007). Based on the lower ploidy variation in sperm nuclei and the higher proportion of seeds receiving a 2*n* male contribution to the endosperm in odd-ploid compared with even-ploid apomictic *Potentilla* species, Dobeš *et al.* (2013) hypothesized that pollen tends to be unreduced in odd-ploids. The present results, however, do not support this idea as odd-ploids received, on average, a higher percentage of *n* male contributions compared with the even-ploid apomicts (based on the assumption of only marginal intercytotype gene flow; Supplementary Data Fig. S2). Alternatively, Müntzing (1928) proposed an increasing number of genomes to alleviate disadvantageous effects of aneuploid chromosome numbers on pollen viability, which probably explains the difference in pollen quality between penta- and heptaploids (Fig. 1). Consequently, pollen quality in the apomictic cytotypes might be governed by the interacting effects of the number of genomes carried by an individual.

## SUPPLEMENTARY DATA

Supplementary data are available online at www.aob.oxfordjournals.org and consist of the following. Results: detailed FCSS results, and the link between the amount of endosperm and the limit on application of FCSS. Figure S1: frequency distribution of peak indices observed in five cytotypes of *Potentilla puberula*. Figure S2: associations of male and female genomic contributions in sexually and apomictically derived seeds in *Potentilla puberula*. Figure S3: flow cytometry of *Potentilla puberula* seeds representing three observed pathways of seed formation defined by a particular combination of embryo ploidy and endosperm/embryo fluorescence ratio. Figure S4: associations between the cytotype diversity and the variation in the male genomic contribution to the embryo of sexually derived seeds formed by tetraploid maternal plants of *Potentilla puberula* as observed in seven populations. Table S1: the cytological diversity of 22 populations of *Potentilla puberula* in East Tyrol, Austria, inferred from Hülber *et al.* (2013). Table S2: generalized linear models comparing the proportion of viable pollen grains between tetraploids and higher polyploids of *Potentilla puberula*.
